# Proteomics—An unexpected journey into the complexity of protein structures and functions

**DOI:** 10.1016/j.euprot.2018.11.001

**Published:** 2018-11-17

**Authors:** Friedrich Lottspeich

**Affiliations:** Am Klopferspitz 4a, D 82131 Stockdorf, Germany

Before 1975, classical protein chemistry was a prime and very successful tool to gain biological and functional knowledge about life science processes. Usually motivated by an observation of a specific biological effect researchers then tried to identify a protein correlated to the functional observation. This reductionistic approach has generated the knowledge, which filled the standard text books on biochemistry and cellular physiology for almost hundred years. Powerful but tedious separation techniques like chromatography and electrophoreses in combination with Edman amino acid sequencing made it possible to enrich, purify and functionally analyze almost any protein – although spending major effort and time and having novel prize winners dedicate a decade of their life to a single protein or biochemical reaction.

## 1 History of proteomics

In 1975 high resolution two dimensional gels, independently invented by O´Farrel and Klose initiated new ways to solve biological questions. Immediately, protein chemists adopting these methods realized that the many proteins, separated and visualized by 2D gel might have the potential to create a new type of bioanalysis. This could be based on a comparison of all proteins in defined biological situations by a kind of differential approach. This holistic concept has in fact been the basis of modern protein chemistry up to now.

However, there was a wide gap between the ambitious dream and reality. Sequence analysis was very slow in the late seventies and not sufficiently sensitive to effectively analyze the ng-amounts of protein in 2D-gels. Furthermore, many technical problems compromised proper sample preparation as well as high quality protein separation by 2D gels. At the same time most promising molecular biology techniques, especially cloning and DNA-sequencing popped up and attracted the interest of almost all young scientists. Conversely, the concept of high throughput approaches were regarded not any more exotic, since in the nucleic acid field – a mainstream research analytical area of the 90th - its feasibility was proven and biological relevant data were generated.

Thus, throughout the late eighties mainly descriptive collections of 2D gel maps can be found in the literature. And when taking a problem solving perspective and looking back to those times, almost all of the results turned out mainly useless. However, concerning instrumental development and bioinformatics it was a very productive period. Especially relevant was mass spectrometry entering the field of proteins and peptides and it got major impact: MALDI-MS was developed and proved that MS is capable of analyzing proteins - but still with major restrictions concerning analytical depth, mass range and quantification. Sample preparation techniques interfacing gel based separations and mass spectrometry were developed.

In the early 90^th^ several facts and insights came together:

• The limit of genomics became apparent. Obviously, molecular biology methods had their own restrictions, especially with respect to an in-depth analysis of molecular functions related to specific genes and their products.
• Genome databases and informatics were growing fast. Due to computer and software development and the internet the databases could be easily accessed and used.
• Electrospray ionization (ESI) was developed and applied to peptide analysis: ESI proved to be able to analyze peptides in a high through-put fashion when coupled to reverse phase HPLC. Algorithms like MASCOT and SEQUEST were developed that allowed to link peptide detection to gene-based databases.
• LC–MS coupling became routine.
• In 1994 the buzz word “proteomics” was born. A fantastic word - suggesting a similarity to genomics and similar potential for success - which switched the old fashioned” protein chemistry” to a new fancy and modern term.
• With the advent of “Proteomics” the concept of a future holistic analyses (complete and accurate) became present and publishable.

This concept created an incredible hype. Academia, industry and politics ran for this “Holy Grail” and invested. Societies were founded and big consortia tried to technically and conceptually realize the holistic dream.

Immediately it became clear that mass spectrometry occupied the driver seat of proteomics. And, as it was evident that large intact proteins were very difficult to analyze, small peptides could easily be analyzed like small molecules via mass spectrometry. And a genius concept, which had never been considered by a protein chemist was realized by mass spectrometric scientists: “cleave your protein(s) into peptides and regard these peptides as surrogates for the protein!”. This concept was immediately accepted by the overwhelming majority of scientists in the field. It was pragmatic and straightforward and the alternatives frustrating, laborious and difficult.

However, reality is sometimes different.

The further development was strictly logical. The mass spectrometry companies put their major developmental capacity in this peptide based approach called “bottom up”. Sample preparation and mass spec analysis was optimized to higher and higher sensitivity and throughput. Proteomic scientist and mass spectrometry companies became experts in “number crunching”: the more proteins are identified, the more reputation could be earned. The biological outcome and impact was secondary, quantitative aspects were completely neglected until almost to the end of the century. However, during this time it became very obvious, that the techniques and strategies available had incredibly improved but - despite major efforts - still were not capable to deliver biological important results. As major reasons for this the incredibly underestimated complexity of biological samples and the dynamic range of protein concentrations of more than 10 orders of magnitude were identified. Only a very few groups working on less complex systems like bacteria or protein complexes succeed in analyzing a biological system in a kind of holistic manner.

As a consequence the hype turned into the exact opposite, frustration in the companies that have spent billions to find new bio markers for diseases, frustration of the funding agencies and frustration of the scientists. Even the president of the HUPO (Rolf Apweiler) at a conference in 2005 expressed the common feeling: “proteomics did not give sufficient back for the buck”. And the proteomics idea never fully recovered from this time. In the middle of the first decade in 21^th^ century slowly the proteomics field realized, that results – as for any analytical technique - are strictly required to be quantitative. Strategic routes and protocols for isotopic labeling or label free techniques for proteomics were developed.

However, most of the labeling techniques were performed in combination with bottom up strategies, and thus the conceptual inherent problems of that strategy remained - i.e. the very common and biological relevant posttranslational modifications, isoforms or truncations of proteins are not at all or at least with extreme difficulty to be elucidated.

Nevertheless, despite the labeling procedures on the protein level gave more accurate quantification, they are more expensive and require sophisticated sample preparation techniques which are problematic in giving true quantitative information. The pragmatism again succeeded and today mostly label free techniques are used.

However, concomitant with the steady improvement of the mass spectrometry, in the last decade other strategies for proteomics got into focus.

Top down proteomics – analyzing intact proteins by mass-spectrometry methods became much more mature and today medium to low complex protein mixtures can successfully be analyzed by mass spectrometry alone.

Targeted proteomics techniques (e.g. SRM, Swath) are probably the most promising approaches addressed today. Quantitatively highly accurate results for individual proteins can be obtained from minute amounts and also from very complex samples like plasma. Many scientists strongly believe that these techniques will enter protein analytics and even clinics and will have the potential to compete in many cases with immunological assays. However, the precondition for targeted proteomics is that the protein to be analyzed is well known, which is not entirely in frame with a holistic approach.

## 2 State of the art and current limitations

Even though the instrumental and methodological progress was really astonishing during the last 30 years, especially with mass spectrometry and informatics as the driving forces, the main limitations of all proteomics strategies are in the enormous complexity and diversity of biological systems at the protein level. This complexity combined with a dynamic range of protein concentrations present in biological systems of more than 11 orders of magnitude, overtaxes the quantitative analytical and the strategic conceptual capabilities available until today.

The scientists of the last century completely underestimated the complexity and diversity of biological systems on all levels. Probably we still are reluctant to accept it, and readily rather neglect it. To a large extent it was proteomics which uncovered the incredible complexity of biological molecules and their interactions in space and time. Indeed, in this respect proteomics has contributed significantly to a deeper and more holistic view of nature.

## 3 Future aspects

There is no question that proteomics techniques are already an indispensable tool in any protein chemical analysis and allows us to look closer, in more detail, more sensitive and especially much faster as ever before to the structure of proteins. This incredible development will certainly proceed and mass spectrometry is today undisputed in the technological driver seat.

However, in my opinion, major obstacles are on the way to fulfill the original dream of the efficient synergy of protein chemistry and systems biology:

• As mentioned, we are confronted with an unexpected degree of complexity and dynamic range of living systems, which I my opinion we have to accept as a fact and start to solve the problem.
• The diversity of higher biological systems, and the influence of the environmental factors on individuals forces us to implement new concepts like individualized proteomics, e.g. to fulfill the requirements of personalized medicine.
• From the proteomics side only little effort or thoughts are put towards the important spatial arrangement of the proteins within a cell, organ, etc. Concepts of quantitative high throughput analysis of posttranslational modifications in context with spatial distribution are almost not available.
• Despite the great methodological progress we are far from being able to analyze biological networks and systems in a holistic manner. A quick change of this situation cannot be envisioned in the near future. The high costs of the analytical instruments (e. g MS) and thus detailed and repetitive statistical significant quantitative analyses are much too expansive and so far spectacular positive biological answers given by proteomics are almost missing. Thus, the trust in the problem solving power of proteomics is rather low.
• Proteomics today is almost never regarded as a new concept to answer biological questions. Today proteomics very often is used as a synonym for high throughput mass spectrometric analysis of proteins, which is much narrower than we intended earlier.

## 4 Outlook

Today we get an eerily beautiful feeling of the complexity and multiplicity of interleaved networks involved in biological systems. Some fundamental misconceptions in the evolution of proteomics and the overly powerful position of mass spectrometry in this field have led to a rather narrow view on the potential of proteomics. Conceptually, proteomics is not only high throughput mass spectrometry. However, in my opinion, to prove proteomics as a truly problem solving science a high degree of innovation and major fundamentally novel technical steps will be needed. Maybe we have to exploit the potential of single protein molecule analysis analogous to nucleic acid techniques. Sample preparation methods compatible with quantitation probably in combination with informatics and high throughput have to be developed. Also the impact of optical methods may be necessary to exploit also the enormous relevant spatial aspects in biology.

I believe that our dream of the comprehensive systematic and holistic protein analysis view, originally named proteomics, is still alive, but certainly will need quantum leaps in concepts and technology and in my opinion there is still a long way to go including investing in solid basic research to reach this goal.

“Today the network of relationships linking the human race to itself and to the rest of the biosphere is so complex that all aspects affect all others to an extraordinary degree. Someone should be studying the whole system, however crudely that has to be done, because no gluing together of partial studies of a complex nonlinear system can give a good idea of the behavior of the whole.”

Murray Gell-Mann

